# The Effect of a mHealth App (KENPO-app) for Specific Health Guidance on Weight Changes in Adults With Obesity and Hypertension: Pilot Randomized Controlled Trial

**DOI:** 10.2196/43236

**Published:** 2023-04-12

**Authors:** Naoki Sakane, Akiko Suganuma, Masayuki Domichi, Shin Sukino, Keiko Abe, Akiyoshi Fujisaki, Ai Kanazawa, Mamiko Sugimoto

**Affiliations:** 1 Division of Preventive Medicine Clinical Research Institute National Hospital Organization Kyoto Medical Center Kyoto Japan; 2 Technology Development HQ OMRON HEALTHCARE Co., Ltd. Kyoto Japan; 3 Cardiovascular Disease Business Planning Strategy HQ OMRON HEALTHCARE Co., Ltd. Kyoto Japan

**Keywords:** obesity, hypertension, mobile health care app, specific health guidance, obese, weight, mHealth, mobile health, mobile app, health app

## Abstract

**Background:**

Commercial smartphone apps that promote self-monitoring of weight loss are widely available. The development of disease-specific apps has begun, but there is no app for specific health guidance (SHG) to prevent metabolic syndrome, type 2 diabetes, and cardiovascular diseases in middle-aged adults in Japan.

**Objective:**

This study aimed to determine the efficacy of an SHG mobile health app in facilitating weight loss in Japanese adults with obesity and hypertension.

**Methods:**

In a 12-week, statistician-blinded, randomized parallel controlled trial, 78 overweight and obese men aged 40-69 years were assigned in a 1:1 ratio to either the usual support plus KENPO-app group (intervention group) or the active control group. KENPO-app (release April 10, 2019; OMRON Healthcare Co., Ltd.) was developed by the study team and focus groups and uses behavior change techniques (ie, self-monitoring and goal-setting theory). This app was developed for SHG based on the four specific health checkups and guidance system in Japan: (1) focusing primarily on achieving the target (weight loss of ≥2 kg); (2) assessing healthy eating, exercise habits, smoking habits, relaxation, and self-weighing; (3) providing information on the results of specific health checkups; and (4) starting an intervention period of 6 months with the interim assessment at 3 months. The initial assessment explored the following: personality traits (4 types), health checkup data concerns (10 items), symptom concerns (10 items), and the aim of the intervention (weight loss, improving fitness, symptoms, laboratory data). Chatbot-supported health information on health and health behavior was selected from 392 quizzes based on app data and was provided to participants. The KENPO-app had chatbot-supported feedback and information provision combined with a self-monitoring tool (weight, steps, and blood pressure). Data on active exercise, healthy eating, and healthy lifestyle habits were obtained using a web-based self-administered questionnaire at baseline and 12 weeks.

**Results:**

The trial’s retention rate was 95% (74/78). The adherence to daily self-weighing, wearing the pedometer, and blood pressure monitoring in the KENPO-app group was significantly higher than those in the active control group. Compared with the active control group, the median body weight and BMI of the intervention group significantly decreased at 3 months (–0.4, IQR –2.0 to 0.6 kg vs –1.1, IQR –2.7 to –0.5 kg; *P*=.03; –0.1, IQR –0.6 to 0.3 kg vs –0.4, IQR –0.8 to –0.2 kg; *P*=.02, respectively). The intervention increased the percentage of participants who self-reported taking ≥8000 steps, eating vegetables before rice, eating slowly, and relaxing. Personality traits were associated with the degree of weight loss in the intervention group.

**Conclusions:**

The SHG-specific KENPO-app was feasible and induced modest but significant weight loss in adults with obesity.

**Trial Registration:**

University Hospital Medical Information Network Center UMIN000046263; https://tinyurl.com/bderys3b

## Introduction

In 2008, all health insurers in Japan were mandated to provide specific health guidance (SHG) to prevent metabolic syndrome, type 2 diabetes, and cardiovascular diseases in middle-aged adults in Japan [1-6]. During the first implementation stage, between 2008 and 2012, the nationwide implementation goal was 45%. However, after reassessment in 2019, the actual implementation rate was far lower at 23.2%. During the second stage, between 2013 and 2017, the flow of the SHG process was reviewed. During the COVID-19 pandemic, self-quarantine was associated with unhealthy eating habits, sedentary behavior, and weight gain [7,8]. In addition, the efficacy of SHG was small, and repeater eligibility for SHG was a problematic issue [9]. During the third stage, between 2019 to 2023, SHG using information and communications technology (ICT) was initially introduced in 2021. For the fourth stage (2024-), the evaluation of the outcome (ie, ≥2 kg of weight loss) will be performed, and a mobile health (mHealth) care app will be introduced for SHG. Overall, research evidence suggests that mobile apps and wearables are effective self-regulating tools for weight loss in the Western population, but a discrepancy exists [10,11]. Commercial smartphone apps that promote the self-monitoring of weight loss are widely available. The development of disease-specific apps has begun. Several apps are used for real-world SHG, but there is no app specified for SHG. Therefore, we developed the SHG-specific mHealth app (KENPO-app), and this study aimed to determine its efficacy in facilitating weight loss in Japanese adults with obesity and hypertension.

## Methods

### Study Design

This study was a 12-week, statistician-blinded, parallel-group, randomized controlled trial (RCT) of adults with obesity and hypertension. The data were obtained between October 2021 and May 2022.

### Ethics Approval

The study protocol was approved by the institutional review board of Kyoto Medical Center (NO.21-057), and the protocol of the study was registered at the University Hospital Medical Information Network Center (UMIN000046263).

### Participants

Participants were recruited from the screening panel of the Omron monitor recruitment site for product development and research in Japan. Therefore, participants may have relatively higher computer/internet literacy. We held an online information session for this trial. Inclusion criteria were as follows: age 40-64 years, BMI≥25 kg/m^2^, systolic blood pressure (SBP) ≥130 mm Hg or diastolic blood pressure (DBP) ≥85 mm Hg, smartphone users capable of installing apps, and individuals capable of communicating online. Exclusion criteria were as follows: receiving SHG at present, taking antihypertensive medicine, contraindication for healthy eating and active exercise by a doctor, pregnant or breastfeeding women, and inappropriate cases (ie, severe psychiatric disorders) as determined by a research doctor.

### Randomization and Masking

The independent statistician randomly allocated participants into one of two intervention arms according to sex-, age-, SBP-, and BMI-stratified block randomization (seed=1221 and block size=2). We adopted a single-blind approach; thus, the effectiveness was assessed by blinded researchers who were unaware of the randomization results.

### Self-monitoring Tool

Participants in both groups received a Bluetooth weighing scale (HBF-227T, OMRON Healthcare Co, Ltd), pedometer (HJA-405T, OMRON Healthcare Co, Ltd), and upper arm blood pressure (BP) monitor (HCR-7501T, OMRON Healthcare Co, Ltd).

### The Active Control Group

The participants in the active control group received the usual support. The usual support was based on intensive SHG, and the participants in both groups received initial online face-to-face counseling by a health care professional (ie, a registered dietician) who had completed the established Ministry of Health, Labor and Welfare (MHLW) training course. Participants were briefed about their health condition and lifestyle through a review of their SHG results. They were instructed to set achievable personalized behavioral goals. After the initial counseling, a health care professional provided email support three times at 2, 6, and 12 weeks. Implementation points according to the MHLW in the active control group were comparable to the required points of ≥180 in the SHG. Daily recording of body weight and steps were recommended. Measurements of BP in the morning and evening were also recommended.

### mHealth KENPO-app

**Figure 1:**
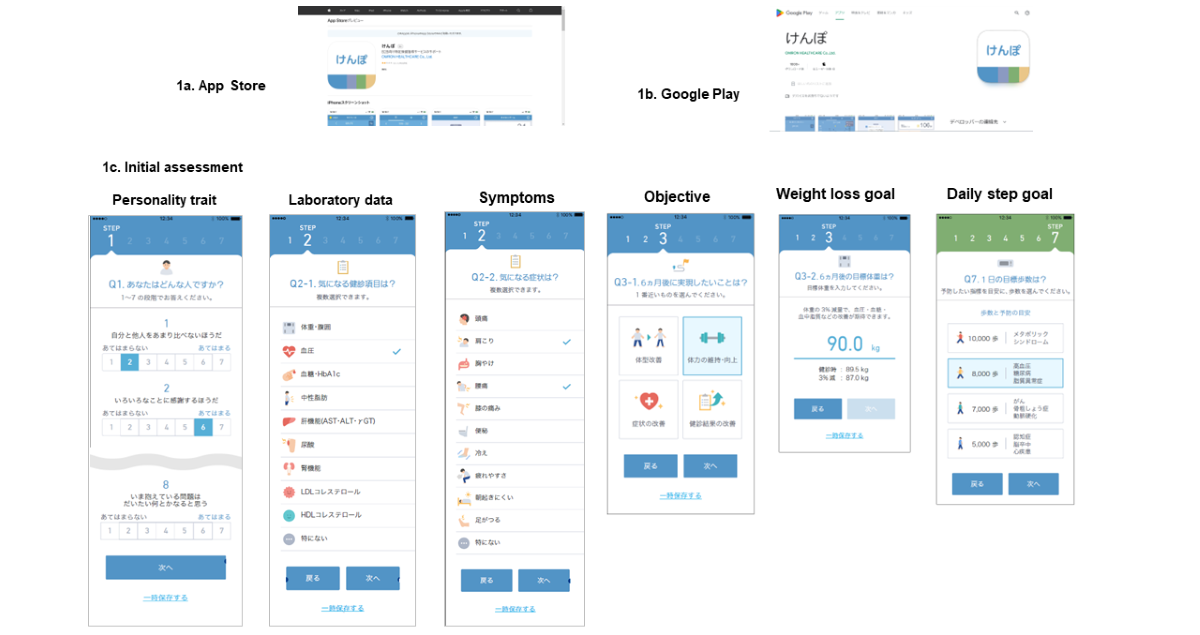
Screenshot and initial assessment of KENPO-app system. (A) KENPO-app at APP Store; (B) KENPO-app at Google Play; (C) initial assessment items.

**Figure 2:**
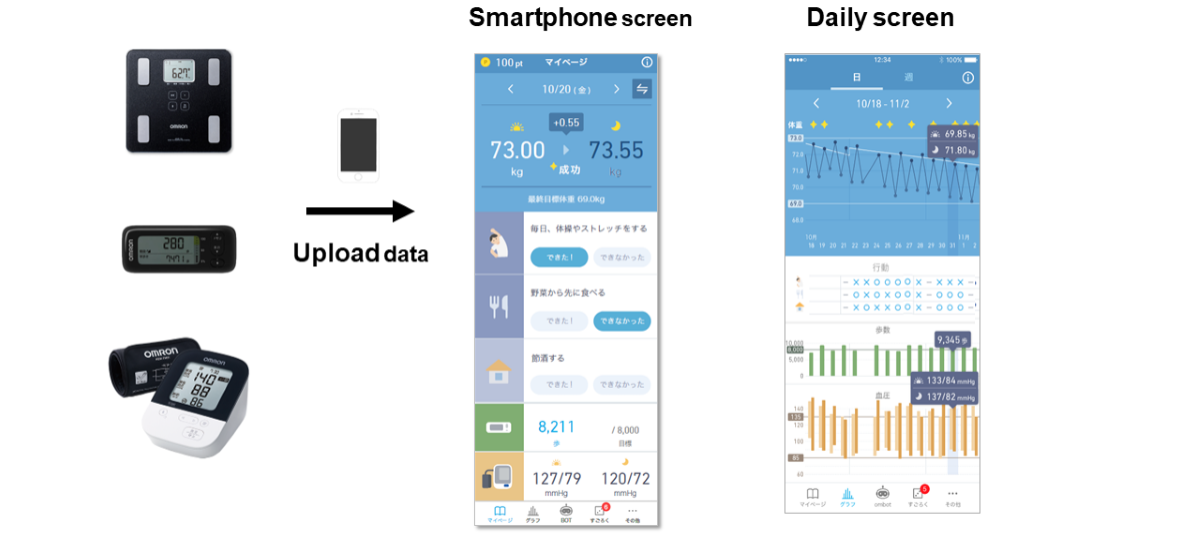
Outline of KENPO-app system.

KENPO-app (release April 10, 2019; OMRON Healthcare Co, Ltd) was developed using behavior change techniques (ie, self-monitoring and goal-setting theory) by the study team and focus groups. This app was developed for SHG based on the four specific health checkups and guidance system in Japan: (1) focusing on achieving the primary target (weight loss of ≥2 kg); (2) assessing healthy eating, exercise habits, smoking habits, relaxation, and self-weighing; (3) providing information on the results of specific health checkups; and (4) starting an intervention period of 6 months with the interim assessment at 3 months. The initial assessment explored the following: personality traits (4 types), concerns about health checkup data (10 items), concerns about symptoms (10 items), and the aim of the intervention (weight loss, improving fitness, symptoms, laboratory data; Figures 1 and 2). Saeki et al [12] reported a cluster analysis that showed 4 clusters in a total of 1500 people aged 15-75 years. They classified personality traits into four types: “Challenger” (self-realization and a sense of growth; fact-based extrovert), “Entertainer” (connection and gratitude; relationship introvert), “Communicator” (optimism; relationship introvert), and “Walker” (do things at my own pace; fact-based introvert). The targeted behavioral goals were as follows: exercise habits (10 items), healthy eating habits (10 items), lifestyle habits (10 items), and daily steps (5000, 7000, 8000, and 10,000 steps). The self-administrated questionnaire on exercise, healthy eating, and lifestyle habits had three choices: “not confident,” “ready to change,” and “already have been.” Chatbot-supported health information on health and health behavior was selected from 392 quizzes based on app data that was provided to participants (Textbox 1). Participants accessed the KENPO-app from the App Store or Google Play. Self-weighing twice was recommended. We did not perform revisions or updates during the study period. Safety and security procedures included privacy considerations and the availability of a hotline.

**The contents of the KENPO-app study.**

**Input to the app**

- Initial assessment (laboratory data, symptoms, and clinical goals)
- Personality trait
- Setting weight loss goal
- Targeting behavioral agenda (exercise, dietary, lifestyle habits, daily steps)

**Self-monitoring (before initial specific health guidance [SHG])**

- Pedometer, weight, and blood pressure, and behavioral agenda

**Online initial SHG**

- SHG based on app data and personality trait

**Self-monitoring (after initial SHG)**

- Pedometer, weight, and blood pressure, and behavioral agenda
- Chatbot-supported feedback of app data and sign of weight regain

**Quiz on health**

- Chatbot-supported quiz on health and health behavior

**Input to the app (12 weeks)**

- Final assessment (laboratory data, symptoms, objective)
- Behavioral agenda (exercise, dietary, and lifestyle habits)

### Outcome

The outcome included changes in body weight and BMI. Weight measurements were uploaded to the cloud where the data were obtained, and the 7-day average weight was calculated. Other outcomes included changes in SBP, DBP, and adherence to the device. The frequency of weight, BP, and step uploads was recorded as a measure of adherence. Data on active exercise habits (10 items), healthy eating habits (10 items), and healthy lifestyle habits (10 items) were obtained using a web-based self-administered questionnaire at baseline and 12 weeks. Quality assurance was performed through standard operating procedures and benchmarking. Adherence to the apps was defined based on the sending rate of body weight measurements, and an attrition diagram was made.

### Statistical Analysis

Data are expressed as the mean (SD), median (IQR), range, or number. Blinded data were analyzed using the R software version 4.0.0. (R Foundation for Statistical Computing) on an intention-to-treat basis by the statistician. Statistical analysis of quantitative data was performed using the Shapiro-Wilk test, Mann-Whitney *U* test, Student *t* test, and Spearman rank test. Categorical data were analyzed using Fisher exact test or exact binomial test. Those cases with missing data were omitted in the relevant analysis. Sensitivity analysis was performed using multiple imputation. There was no previous study related to our hypothesis. Therefore, the sample size was estimated as 52 people with an effect size of 0.8 (large effect size) for a pilot or feasibility study. With a dropout rate of approximately 30%, 80 people were required. The level of statistical significance was set at *P*<.05.

## Results

### Participants and Adherence

**Figure 3:**
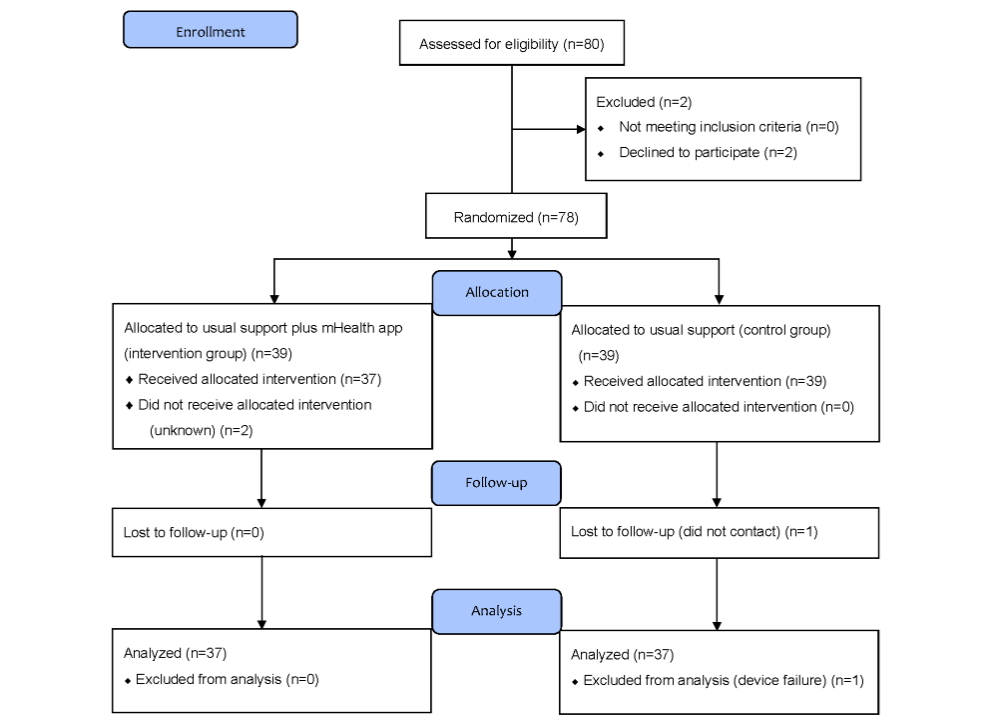
CONSORT flow diagram of KENPO-app study.

**Figure 4:**
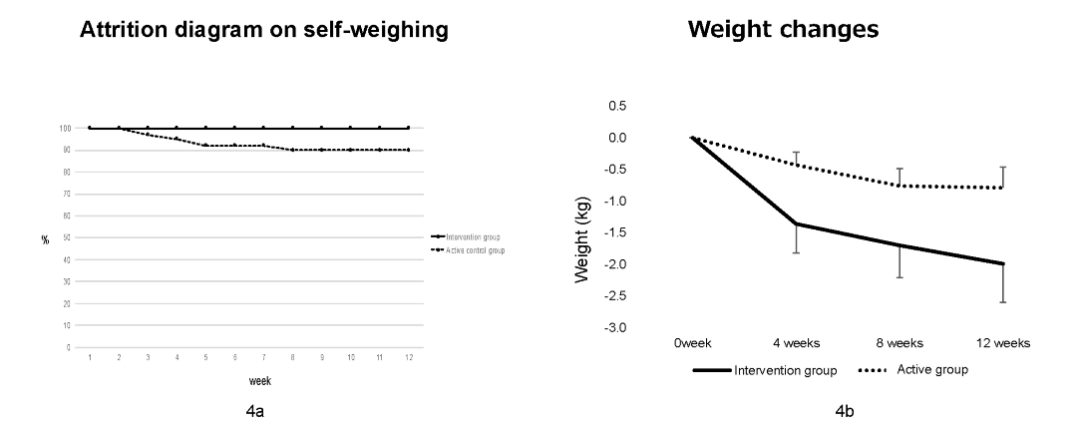
Attrition diagram on self-weighing and mean changes in body weight during the study period.

After 80 participants were screened, we enrolled 78 participants and excluded 2. Of the 78 participants, the mean age was 52.0 (SD 6.5) years, and 55% (n=43) were male. Those who had full- and part-time jobs accounted for 65% (n=51) and 19% (n=15) of the sample, respectively. Participants were assigned to either the KENPO-app group (intervention group; n=39) or the active control group (n=39). There were no differences in age (mean 52.5, SD 6.6 years vs mean 51.4, SD 6.4 years; *P*=.47), male sex ratio (56.4% vs 53.8%; *P*>.99), BMI (mean 27.5, SD 1.9 km/m2 vs mean 27.9, SD 2.3 kg/m2; *P*=.41), or SBP (mean 138.3, SD 12.7 mm Hg vs mean 136.2, SD 17.5 mm Hg; *P*=.55) between the groups. Two participants in the intervention group did not receive the intervention before the initial web-based consultation. We could not contact 1 participant after the intervention for unknown reasons in the active control group. As indicated in the CONSORT flow diagram, one participant was deleted from the analysis due to device failures in the active control group (Figure 3). The trial retention was 95% (74/78). Figure 4A shows the self-weighing attrition diagram. The adherence to daily self-weighing, wearing the pedometer, and BP monitoring in the KENPO-app group was significantly higher than those in the active control group. Participants in the KENPO-app group had the following health checkup data concerns: hyperglycemia 27% (n=10), hypertriglyceridemia 57% (n=21), and low high-density lipoprotein cholesterol 24% (n=9).

### Outcome

Figure 4 shows the weight changes during the study (mean –2.0, SD 0.6 kg in the intervention group vs mean –0.8, SD 0.3 kg in the active control group at the 12-week follow-up). The distribution of the weight changes was not normal as assessed by the Shapiro-Wilk test. Compared with the active control group, the median body weight and BMI of the intervention group significantly decreased at 3 months (–0.4, IQR –2.0 to 0.6 kg vs –1.1, IQR –2.7 to –0.5 kg; *P*=.03; –0.1, IQR –0.6 to 0.3 kg vs mean –0.4, IQR –0.8 to –0.2 kg; *P*=.02, respectively). The sensitivity analysis confirmed the results. The odds ratio for achieving ≥3% and 2% weight loss was 1.58 (95% CI 0.47-5.63) and 2.27 (95% CI 0.79-6.85), respectively. Personality traits were associated with the degree of weight loss in the KENPO-app group. Compared with “challenger” (n=7), “walker” (n=6) had significantly greater weight loss (median –0.50, IQR –0.65 to 0.40 kg vs median –3.10, IQR –4.42 to –2.00; *P*=.02), but there was no difference in weight change among “challenger,” “communicator,” and “entertainer.” The adherence to daily self-weighing and BP monitoring in the intervention group was significantly higher than in the active control group (daily self-weighing: mean 79.3%, SD 10.5% vs mean 68.6%, SD 22.1%; *P*=.01; BP monitoring: mean 78.0%, SD 11.4% vs mean 58.8%, SD 31.6%; *P*=.001, respectively). Similarly, adherence to daily steps was higher in the intervention group than in the active control group (mean 75.7%, SD 13.4% vs mean 68.2%; *P*=.09). Only adherence to self-weighing in the morning and evening was negatively correlated with changes in body weight in the intervention group, although adherence to daily self-weighing and daily steps in both groups was not. SBP decreased in the intervention group (from mean 138, SD 13 mm Hg to mean 135, SD 10 mm Hg; *P*=.02), but this was not significantly different from the active control group. There were no changes in DBP observed in the intervention group.

### Healthy Behavior

The percentage of participants who reported ≥8000 steps per day, slow eating speed, vegetable intake before rice, selecting a healthy menu, and relaxation in the intervention group increased after the 12-week study period, while the percentage of eating breakfast and reducing snacks in the active control group increased (Table 1). The rate of achieving ≥8000 steps based on the pedometer after the intervention tended to be higher than that in the active control group (58.8% vs 32.4%; *P*=.05). There were no severe adverse events or technical problems.

**Table 1:** Healthy lifestyle and symptoms during the study period according to the group.

Item			Intervention group (n=39), n (%)							Control group (n=39), n (%)				
			Pre		Post		*P* value		Pre			Post		*P* value
**Active exercise habits**														
	Use of stairs instead of the escalators	5 (14)		8 (22)		.45		9 (24)			14 (38)		.13	
	Walking ≥8000 steps	6 (16)		13 (35)		.046		9 (24)			8 (22)		>.99	
	At least 30 min of brisk daily walks	4 (11)		9 (24)		.18		6 (16)			6 (16)		>.99	
	Do gymnastics/stretching everyday	3 (8)		6 (16)		.37		2 (5)			8 (22)		.08	
	Do not stay home and do nothing on holiday	3 (8)		7 (19)		.22		7 (19)			12 (32)		.18	
	Stand up and exercise once an hour	8 (22)		13 (35)		.18		9 (24)			9 (24)		>.99	
	Do housework (cooking, cleaning, etc)	15 (41)		18 (49)		.37		24 (65)			25 (68)		>.99	
	Resistance training ≥3 times per week	1 (3)		2 (5)		>.99		1 (3)			2 (5)		>.99	
	Use the gym or pool at least once per week	1 (3)		1 (3)		>.99		0 (0)			1 (3)		>.99	
	Play sports at least once a week	0 (0)		4 (11)		.13		3 (8)			4 (11)		>.99	
**Healthy eating habits**														
	Eat moderately	1 (3)		7 (19)		.08		6 (16)			10 (27)		.22	
	Eat breakfast	22 (60)		22 (60)		>.99		25 (68)			31 (84)		.04	
	Eat vegetable first	12 (32)		23 (62)		.01		22 (60)			26 (70)		.22	
	Eat slowly and well	1 (3)		9 (24)		.01		13 (35)			16 (43)		.37	
	Eat with nutritional balance in mind	4 (11)		9 (24)		.13		12 (32)			15 (41)		.45	
	Do not overeat carbohydrates	1 (3)		5 (14)		.13		9 (24)			14 (38)		.13	
	Eat fried food up to 3 times per week	1 (3)		6 (16)		.07		12 (32)			16 (43)		.29	
	Reduce salt	1 (3)		6 (16)		.07		11 (30)			12 (32)		>.99	
	Reduce sweet buns and delicatessen bread	7 (19)		9 (24)		.72		11 (30)			14 (38)		.37	
	Reduce eating in dinner	1 (3)		4 (11)		.25		8 (22)			13 (35)		.13	
**Healthy lifestyle**														
	Reduce sugar-sweetened beverages	8 (22)		14 (38)		.15		20 (54)			23 (62)		.45	
	Reduce sweets	4 (11)		5 (14)		>.99		5 (14)			15 (41)		.02	
	Check food labels	7 (19)		12 (32)		.13		11 (30)			14 (38)		.45	
	Choose healthy menu	0 (0)		8 (19)		.02		9 (24)			9 (24)		>.99	
	Do not eat anything 2 h before bedtime	9 (24)		15 (41)		.08		13 (35)			14 (38)		>.99	
	Go to bed early	8 (22)		12 (32)		.39		10 (27)			10 (27)		>.99	
	Try to have alcohol-free days	26 (70)		30 (81)		.29		27 (73)			29 (78)		.62	
	Reduce alcohol drinks	27 (73)		28 (76)		>.99		24 (65)			25 (68)		>.99	
	Stop smoking	33 (89)		32 (87)		>.99		33 (89)			33 (89)		>.99	
	Relaxation	6 (16)		16 (43)		.02		15 (41)			13 (35)		.79	
**Subjective symptoms**														
	Headache	10 (27)		3 (8)		.07		8 (22)			7 (19)		>.99	
	Shoulder stiffness	23 (62)		12 (32)		.006		21 (57)			13 (35)		.04	
	Heartburn	6 (16)		1 (3)		.07		7 (19)			1 (3)		.04	
	Lumbago	17 (46)		9 (24)		.10		17 (46)			15 (41)		.75	
	Knee pain	13 (35)		8 (22)		.12		6 (16)			5 (14)		>.99	
	Constipation	7 (19)		0 (0)		.02		8 (22)			8 (22)		>.99	
	Chillness	9 (24)		2 (5)		.02		13 (35)			4 (11)		.02	
	Fatigue	11 (30)		8 (22)		.37		14 (38)			10 (27)		.29	
	Difficulty in waking up	3 (8)		3 (8)		>.99		9 (24)			7 (19)		.68	
	Leg cramps	6 (16)		2 (5)		.13		6 (16)			5 (14)		>.99	

## Discussion

### Principal Findings

This is the first study to confirm the effectiveness of the SHG-specific KENPO-app in obese adults with hypertension. The mHealth KENPO-app is feasible and can produce modest but significant weight loss. Compared with standard care, the mHealth app produced modest weight loss (–1.0 kg to –2.4 kg of body weight) in obese adults with diabetes [13]. The meta-analysis by Ang et al [14], including 17 articles for Asian populations, indicated that the effect size of the RCTs for weight change was small to moderate. The effect size of our results was also small to moderate. In this study, we observed a significant change in SBP. Although mHealth apps are effective in reducing weight, they were ineffective in lowering BP in 160 adults with ≥2 cardiovascular risk factors [15]. Further examination, including a large sample size, is required to confirm these issues in the future.

### Adherence and Weight Change

Self-monitoring of weight was a significant predictor of weight loss [16-18]. In this study, self-weighing twice a day (in the morning and evening) was negatively correlated with weight change, although daily self-weighing was not. We previously reported that self-weighing twice per day plus daily target setting and feedback were more effective in promoting weight loss than one daily self-measurement [19]. Self-weighing twice a day is a recommendation in clinical practice.

### Healthy Behavior and Weight Loss

The current consensus states that obtaining less than 5000 steps per day is sedentary behavior, whereas obtaining >8000 steps indicates an active exercise habit [20]. A meta-analysis by Flores Mateo et al [21] indicated that the mHealth app was associated with significant changes in body weight and BMI of –1.04 kg and –0.43 kg/m^2^, respectively, compared with the control group. However, there was no significant difference in physical activity between the groups. On the other hand, Richardson et al [22] performed a meta-analysis on pedometer-based interventions without caloric restriction, with a pooled estimated change in body weight of −1.3 kg. In this study, the KENPO-app increased the proportion of ≥8000 steps (self-reported).

Moreover, slow eating speed, vegetable intake before rice, selection of a healthy menu, and relaxation in the intervention group increased after the intervention. Fast eating speed is positively associated with obesity [23,24]. For weight loss strategies, the recommendation of increased vegetable consumption is often used [25]. Meal sequence, such as eating vegetables before rice, reduces postprandial glycemic and weight loss effects [26]. In a meta-analysis by Tapsell et al [27], 5 participants reported greater weight loss, 9 reported no difference, 1 showed weight gain, and 1 reported a positive association between weight loss and high vegetable consumption. Comprehensive healthy eating may have resulted in significant weight loss in the study.

### Personality Traits and Weight Changes

Personality traits are an important factor in health behaviors. Interestingly, personality traits were associated with the degree of weight change in this study. Specific aspects of personality (ie, agreeableness) are relevant to weight loss maintenance [28,29]. People with greater openness and conscientiousness were associated with greater compliance with self-care [30]. Personality traits such as neuroticism, agreeableness, and conscientiousness are associated with self-weighing frequency, dietary habits, support, and difficulties during the weight loss process [31]. Further examinations including the big five personality traits (neuroticism, extraversion, openness, agreeableness, and conscientiousness) and large sample sizes are required to confirm these issues in the future.

### Strength and Limitations

The strengths of this study include the SHG-specific mHealth app, objective measurement of data, and a high retention rate. Although mHealth apps for weight management are popular and widely available, many apps lack professional content expertise. Encouraging app development based on evidence-based online approaches would ensure content quality, allowing health care professionals to recommend their use [32]. However, there are several limitations, including the short-term (12 weeks) and lack of laboratory data. Careful attention should be paid to interpretations regarding the results because of the lack of blinding. In this study, health and ICT literacy may be higher compared to participants in the real-world needing SHG. We did not analyze the cost-effectiveness. Further examinations including cost-effective analysis are required to confirm these issues in real-world SHG. The generalizability of the findings is limited to other populations due to it being in the Japanese language and being an SHG-specific app.

In conclusion, the SHG-specific KENPO-app was feasible and induced significant weight loss in Japanese adults with obesity and hypertension.

## Appendix

Multimedia Appendix 1 CONSORT-eHEALTH checklist (V 1.6.1).

## Abbreviations

BP: blood pressure
DBP: diastolic blood pressure
ICT: information and communications technology
mHealth: mobile health
MHLW: Ministry of Health, Labor and Welfare
RCT: randomized controlled trial
SBP: systolic blood pressure
SHG: specific health guidance

## References

[1] Tsushita K, S Hosler A, Miura K, Ito Y, Fukuda T, Kitamura A, Tatara K (2018). Rationale and descriptive analysis of specific health guidance: the Nationwide Lifestyle Intervention Program targeting metabolic syndrome in Japan. J Atheroscler Thromb.

[2] Ichikawa D, Saito T, Oyama H (2017). Impact of predicting health-guidance candidates using massive health check-up data: a data-driven analysis. Int J Med Inform.

[3] Nakao YM, Miyamoto Y, Ueshima K, Nakao K, Nakai M, Nishimura K, Yasuno S, Hosoda K, Ogawa Y, Itoh H, Ogawa H, Kangawa K, Nakao K (2018). Effectiveness of nationwide screening and lifestyle intervention for abdominal obesity and cardiometabolic risks in Japan: The metabolic syndrome and comprehensive lifestyle intervention study on nationwide database in Japan (MetS ACTION-J study). PLoS One.

[4] Muramoto A, Matsushita M, Kato A, Yamamoto N, Koike G, Nakamura M, Numata T, Tamakoshi A, Tsushita K (2014). Three percent weight reduction is the minimum requirement to improve health hazards in obese and overweight people in Japan. Obes Res Clin Pract.

[5] Enomoto N, Nakamura S, Kanda S, Endo H, Yamada E, Kobayashi S, Kido M, Inoue R, Shimakura J, Narimatsu H (2021). Efficacy of additional intervention to the specific health guidance in Japan: The Takahata GENKI Project. Risk Manag Healthc Policy.

[6] Iseki K, Konta T, Asahi K, Yamagata K, Fujimoto S, Tsuruya K, Narita I, Kasahara M, Shibagaki Y, Moriyama T, Kondo M, Iseki C, Watanabe T (2020). Impact of metabolic syndrome on the mortality rate among participants in a specific health check and guidance program in Japan. Intern Med.

[7] Zachary Z, Brianna F, Brianna L, Garrett P, Jade W, Alyssa D, Mikayla K (2020). Self-quarantine and weight gain related risk factors during the COVID-19 pandemic. Obes Res Clin Pract.

[8] Zeigler Z (2021). COVID-19 self-quarantine and weight gain risk factors in adults. Curr Obes Rep.

[9] Fukuma S, Iizuka T, Ikenoue T, Tsugawa Y (2020). Association of the National Health Guidance Intervention for Obesity and Cardiovascular Risks With Health Outcomes Among Japanese Men. JAMA Intern Med.

[10] Chin SO, Keum C, Woo J, Park J, Choi HJ, Woo J, Rhee SY (2016). Successful weight reduction and maintenance by using a smartphone application in those with overweight and obesity. Sci Rep.

[11] Wang E, Abrahamson K, Liu PJ, Ahmed A (2020). Can mobile technology improve weight loss in overweight adults? A systematic review. West J Nurs Res.

[12] Saeki M, Hasunuma R, Maeno T (2012). The relationships among psychological factors on subjective well-being. Proc Annu Convention JPA.

[13] Wang Y, Min J, Khuri J, Xue H, Xie B, A Kaminsky L, J Cheskin L (2020). Effectiveness of mobile health interventions on diabetes and obesity treatment and management: systematic review of systematic reviews. JMIR Mhealth Uhealth.

[14] Ang SM, Chen J, Liew JH, Johal J, Dan YY, Allman-Farinelli M, Lim SL (2021). Efficacy of interventions that incorporate mobile apps in facilitating weight loss and health behavior change in the Asian population: systematic review and meta-analysis. J Med Internet Res.

[15] Cho SMJ, Lee JH, Shim J, Yeom H, Lee SJ, Jeon YW, Kim HC (2020). Effect of smartphone-based lifestyle coaching app on community-dwelling population with moderate metabolic abnormalities: randomized controlled trial. J Med Internet Res.

[16] Painter SL, Ahmed R, Hill JO, Kushner RF, Lindquist R, Brunning S, Margulies A (2017). What matters in weight loss? An in-depth analysis of self-monitoring. J Med Internet Res.

[17] Han M, Rhee SY (2021). Effectiveness of mobile health applications for 5% body weight reduction in obese and overweight adults. J Obes Metab Syndr.

[18] Sakane N, Oshima Y, Kotani K, Suganuma A, Nirengi S, Takahashi K, Sato J, Suzuki S, Izumi K, Kato M, Noda M, Kuzuya H (2020). Self-weighing frequency and the incidence of type 2 diabetes: post hoc analysis of a cluster-randomized controlled trial. BMC Res Notes.

[19] Oshima Y, Matsuoka Y, Sakane N (2013). Effect of weight-loss program using self-weighing twice a day and feedback in overweight and obese subject: a randomized controlled trial. Obes Res Clin Pract.

[20] Swift DL, Johannsen NM, Lavie CJ, Earnest CP, Church TS (2014). The role of exercise and physical activity in weight loss and maintenance. Prog Cardiovasc Dis.

[21] Flores Mateo G, Granado-Font E, Ferré-Grau C, Montaña-Carreras X (2015). Mobile phone apps to promote weight loss and increase physical activity: a systematic review and meta-analysis. J Med Internet Res.

[22] Richardson CR, Newton TL, Abraham JJ, Sen A, Jimbo M, Swartz AM (2008). A meta-analysis of pedometer-based walking interventions and weight loss. Ann Fam Med.

[23] Zhang T, Cai L, Ma L, Jing J, Chen Y, Ma J (2016). The prevalence of obesity and influence of early life and behavioral factors on obesity in Chinese children in Guangzhou. BMC Public Health.

[24] Kolay E, Bykowska-Derda A, Abdulsamad S, Kaluzna M, Samarzewska K, Ruchala M, Czlapka-Matyasik M (2021). Self-reported eating speed is associated with indicators of obesity in adults: a systematic review and meta-analysis. Healthcare (Basel).

[25] Paixão C, Dias CM, Jorge R, Carraça EV, Yannakoulia M, de Zwaan M, Soini S, Hill JO, Teixeira PJ, Santos I (2020). Successful weight loss maintenance: a systematic review of weight control registries. Obes Rev.

[26] Kubota S, Liu Y, Iizuka K, Kuwata H, Seino Y, Yabe D (2020). A review of recent findings on meal sequence: an attractive dietary approach to prevention and management of type 2 diabetes. Nutrients.

[27] Tapsell LC, Dunning A, Warensjo E, Lyons-Wall P, Dehlsen K (2014). Effects of vegetable consumption on weight loss: a review of the evidence with implications for design of randomized controlled trials. Crit Rev Food Sci Nutr.

[28] Koutras Y, Chrysostomou S, Giannakou K, Kosmidis MH, Yannakoulia M (2021). Personality traits and weight loss maintenance: a cross-sectional study. Front Nutr.

[29] Sullivan S, Cloninger CR, Przybeck TR, Klein S (2007). Personality characteristics in obesity and relationship with successful weight loss. Int J Obes (Lond).

[30] Mendoza-Catalán G, Rodríguez-Santamaría Y, Domínguez-Chávez CJ, Juárez-Medina LL, Villa-Rueda AA, González-Ramírez J, Gutiérrez-Valverde JM (2022). Personality traits and self-care behaviors in adults with type 2 diabetes mellitus. Diabetes Metab Syndr Obes.

[31] Soini S, Mustajoki P, Eriksson JG, Lahti J (2018). Personality traits associated with weight maintenance among successful weight losers. Am J Health Behav.

[32] Nikolaou CK, Lean MEJ (2017). Mobile applications for obesity and weight management: current market characteristics. Int J Obes (Lond).

